# Targeting MDM2 in malignancies is a promising strategy for overcoming resistance to anticancer immunotherapy

**DOI:** 10.1186/s12929-024-01004-x

**Published:** 2024-01-29

**Authors:** Dantong Sun, Haili Qian, Junling Li, Puyuan Xing

**Affiliations:** 1 Department of Medical Oncology, National Cancer Center/National Clinical Research Center for Cancer/Cancer Hospital, Chinese Academy of Medical Sciences and Peking Union Medical College, Beijing, 100021 China; 2 State Key Laboratory of Molecular Oncology, National Cancer Center/National Clinical Research Center for Cancer/Cancer Hospital, Chinese Academy of Medical Sciences and Peking Union Medical College, Beijing, 100021 China

**Keywords:** Malignancies, MDM2, Anticancer immunotherapy, Tumor immune microenvironment

## Abstract

MDM2 has been established as a biomarker indicating poor prognosis for individuals undergoing immune checkpoint inhibitor (ICI) treatment for different malignancies by various pancancer studies. Specifically, patients who have MDM2 amplification are vulnerable to the development of hyperprogressive disease (HPD) following anticancer immunotherapy, resulting in marked deleterious effects on survival rates. The mechanism of MDM2 involves its role as an oncogene during the development of malignancy, and MDM2 can promote both metastasis and tumor cell proliferation, which indirectly leads to disease progression. Moreover, MDM2 is vitally involved in modifying the tumor immune microenvironment (TIME) as well as in influencing immune cells, eventually facilitating immune evasion and tolerance. Encouragingly, various MDM2 inhibitors have exhibited efficacy in relieving the TIME suppression caused by MDM2. These results demonstrate the prospects for breakthroughs in combination therapy using MDM2 inhibitors and anticancer immunotherapy.

## Introduction

The considerable prevalence of malignancies and the resulting high morbidity and mortality present substantial public health concerns. A recent study revealed that the collective number of cancer-related fatalities in 2014 totaled 2,205,200 (including 1,425,700 men and 779,500 women), constituting 22.40% of all deaths occurring in China that year [1]. Fortunately, there have been important advancements in novel therapeutics for cancer treatment in recent years. These advancements include the development of neonatal agents for anticancer targeted therapy and immunotherapy, both of which have greatly benefitted patients. Of particular note are immune checkpoint inhibitors (ICIs), namely, anti-programmed cell death (ligand) 1 [anti-PD(L)1] therapy, which has become the standard therapy for malignancies over the past decade [2]. Several clinical trials have highlighted the valuable role of ICIs in improving the overall survival (OS) of cancer patients [3–7]. However, a limited number of patients show response to this treatment, with only 20–30% of patients benefiting from ICIs [8–10]. Recent reports indicate that a subset of patients (7–29%) might develop hyperprogressive disease (HPD), which is characterized by accelerated tumor growth after immunotherapy [11]. Despite extensive research in this field, the mechanisms underlying resistance to anticancer immunotherapy and HPD remain unclear. The PI3K/Akt signaling pathway is one potential cause of immunotherapy resistance, as reported in some studies [11, 12], but its role is not fully understood. Patients with certain driver mutations, such as epidermal growth factor receptor (EGFR) mutations and anaplastic lymphoma kinase (ALK) rearrangement in non-small cell lung cancer (NSCLC), show poor response to ICIs and may even develop HPD [13, 14]. This is thought to be due to the elevated expression of PD-1, exhaustion of infiltrated immune cells, and suppression of the tumor immune microenvironment (TIME) [15–17]. Furthermore, a previous study suggested a link between anticancer immunotherapy resistance and MDM2, but the underlying mechanism requires further investigation.

The MDM2 gene encodes a 90 kDa protein that acts as a negative regulator of the p53 protein. MDM2 forms the MDM2-p53 complex by binding to p53 through its N-terminal domain (Region I). This interaction leads to the concealment of the p53 transcription activation region, eventually resulting in reduced p53 transcription activity. Furthermore, the ring finger domain of MDM2 functions as an E3 ligase, causing the ubiquitination of p53. Ultimately, the degradation of p53 protein ensues through proteasome interaction. It was believed that MDM2 played a role in the development of resistance to multiple drugs via a mechanism that may have been dependent or independent of p53. The previous review focused on the role of MDM2 in inducing resistance to EGFR-tyrosine kinase inhibitors (TKIs) [1], and then the published research suggested that MDM2 overexpression contributes to resistance to first generation EGFR-TKIs, and EGFR mutant lung adenocarcinoma (LUAD) patients with MDM2 amplification experience poor progression-free survival (PFS) following the administration of these inhibitors [18]. Since then, it has been observed through several studies that MDM2 is correlated with resistance to anticancer immunotherapy. However, the underlying mechanism of this association requires further investigation. The present review highlights the role that MDM2 may serve as a valuable biomarker for anticancer immunotherapy. We provide evidence to support our claim that MDM2 gene amplification is highly correlated with poor prognosis and can even lead to the development of HPD. Through a comprehensive examination of the relevant literature, we explored the underlying mechanisms that contribute to the MDM2-induced poor prognosis that is associated with anticancer immunotherapy. In addition to its oncogenic function, MDM2 can modulate the TIME, which consequently leads to immune tolerance and tumor evasion. Intriguingly, it demonstrated that MDM2 inhibitors can potentially mitigate the suppression of the TIME and have considerable synergistic effects on anticancer immunotherapy.

### MDM2 amplification is associated with a poor reaction to ICIs and may even contribute to the manifestation of HPD in patients with malignancies

In previous studies, we established the potential role of the MDM2 protein, as well as genomic alterations of the MDM2 gene, in the poor prognosis associated with ICI treatment [1, 11]. One common type of MDM2 alteration is its amplification, which often results in MDM2 protein overexpression and is observed in various malignancies, and the incidence of MDM2 amplification ranges from 0% (anaplastic and papillary carcinoma of thyroid) to 63.6% (liposarcoma) [19]. Two pancancer studies revealed that the amplification of MDM2 and its family member MDM4 (MDM2/MDM4 amplification) may serve as a marker for poor response to immunotherapy and HPD [20, 21]. It was observed that patients with MDM2/MDM4 amplification had significantly shorter OS than patients without MDM2/MDM4 amplification (11 months versus 17 months, *P* = 0.0018) after ICI treatment, while there was no significant difference in OS after non-ICI treatment between the two groups of patients [21]. This evidence points to the predictive value of MDM2/MDM4 amplification in the adverse outcomes of ICI treatment. Another pancancer study encompassing six patients with MDM2/MDM4 amplification indicated that all six experienced a time-to-treatment-failure (TTF) of less than two months. Notably, four of the six exhibited an increasedtumor size, the development of new large masses, and an acceleration in tumor progression that was considered HPD following anticancer immunotherapy treatment [20]. To further explore the connection between MDM2 and HPD with regard to anticancer immunotherapy, we reviewed and compiled relevant cases reported in published studies [19, 20, 22], as displayed in Table 1. After conducting a thorough literature review, it was determined that seven patients with MDM2 amplification experienced HPD after receiving ICI treatment. Interestingly, despite the differences in cancer types (such as bladder carcinoma, triple-negative breast cancer, endometrial stromal sarcoma, lung adenocarcinoma, and gastroesophageal junction adenocarcinoma), ICI types (including PD1 and PD-L1 blockade monotherapy), and treatment lines (first-line or nonfirst-line ICI treatment), none of the patients with MDM2 amplification responded positively to ICIs. Instead, their condition rapidly deteriorated, which was characterized by expanded primary lesions, the emergence of new lesions or metastasis, and severe symptoms. Nevertheless, further study is necessary to fully understand the underlying mechanisms that link MDM2 to insensitivity to ICIs.

**Table 1:** Information for patients harboring MDM2 amplification who experienced hyperprogressive disease after anticancer immunotherapy

References	Case	Age	Sex	Diagnosis	Stage	MDM2 amplification	MDM4 amplification	Other genomic alterations	Anticancer immunotherapy (TTF)	HPD diagnosis
Kato et al. Clin Cancer Res. 2017. [20]	1	73	Male	Bladder carcinoma	IV	+	−	−	3 L PD-L1 blockade monotherapy (1.9 m)	Enlargement of existing metastatic lesion (258%), development of new liver metastasis, TTF < 2 months
	2	44	Female	Triple-negative breast cancer	IV	+	−	−	PD-1 blockade monotherapy (1.5 m)	Enlargement of existing metastatic lesion (55%), development of new metastatic lymphnodes, TTF < 2 months
	3	65	Female	Endometrial stromal sarcoma	IV	+	−	−	2 L PD-1 blockade monotherapy (1.5 m)	Enlargement of existing metastatic lesion (242%), TTF < 2 months
	4	50	Female	Lung adenocarcinoma	IV	+	−	KIF5B-RET fusion	2 L PD-1 blockade monotherapy (9 d)	Enlargement of existing metastatic lesion (135%), TTF < 2 months
	5	61	Male	Lung adenocarcinoma	IV	+	−	CDK4 amplification	2 L PD-1 blockade monotherapy (1.5 m)	Development of new brain metastasis, TTF < 2 months
Kato et al. JCO Precis Oncol. 2018. [19]	6	36	Female	Gastro-esophageal junction adenocarcinoma	IV	+	−	Amplifications in ERBB3, ARAF, CDK4, and EGFR; alterations in PIK3CA, FRS2, GLI1, and IKZF1	3 L PD-1 blockade monotherapy (0.75 m)	Enlargement of existing metastatic lesion (460%), development of new metastatic lymphnodes, TTF < 2 months
Mao et al. Drug Des Devel Ther. 2019. [22]	7	58	Male	Bladder carcinoma	IV	+	−	−	1 L PD-L1 blockade monotherapy (1 m)	Enlargement of existing metastatic lesion (1004%), development of new metastatic lymphnodes, TTF < 2 months

### MDM2 has been found to contribute to the development of resistance to ICIs and even HPD through the coordination of various mechanisms

#### The oncogenic function of MDM2 plays an important role in facilitating the proliferation and metastasis of malignancies

**Fig. 1:**
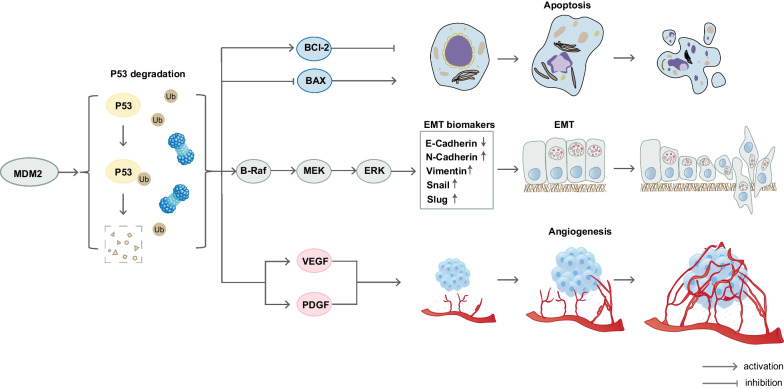
The oncogenic function of MDM2. MDM2 facilitates the degradation of P53, consequently regulating apoptosis inhibition, EMT acceleration, and angiogenesis stimulation in various malignancies

As the biological regulator of P53, MDM2 plays a crucial role as an E3 ligase in enhancing the degradation of the P53 protein through the ubiquitin proteasome system [23]. Furthermore, MDM2 has the ability to eliminate the P53 protein directly from tumor cells [24]. This function enables MDM2 to exert its oncogenic effect that leads to the evasion of cell apoptosis that is typically induced by P53 [25]. In addition to its P53-dependent mode of affecting tumor cell apoptosis, MDM2 can directly modulate the balance of various apoptosis-related molecules, including antiapoptotic molecules such as Bcl-2 and Bcl-xL, as well as apoptotic molecules such as Bax [26, 27]. Consequently, MDM2 can manipulate the balance of these molecules to shift the cell toward an anti-apoptotic state. The process of epithelial to mesenchymal transition (EMT) is widely believed to act as a trigger for cancer metastasis, as it empowers cells to migrate and invade the stroma [28]. Research suggests that MDM2 can stimulate the EMT process by enhancing the expression of EMT-related transcription factors through the activation of the B-Raf signaling pathway [29]. As a result, this process can promote the metastasis of tumor cells, thereby compromising the effectiveness of treatment modalities such as anticancer immunotherapy. Regarding tumor angiogenesis, this phenomenon facilitates the infiltration of tumor cells into the stroma and their subsequent metastasis to distant regions [1]. As early as 1996, studies utilizing immunohistochemistry (IHC) demonstrated that MDM2-overexpressing breast cancer cells exhibited high expression levels of vascular endothelial growth factor (VEGF) and platelet-derived endothelial growth factor (PDGF), indicating the possible involvement of MDM2 in the angiogenesis process of malignancies [30]. Similarly, recent research findings suggest that MDM2 overexpression promotes angiogenesis by inducing a balance in cytokine expression that is conducive to an angiogenic state, ultimately enhancing tumor cell migration and invasiveness [31]. The use of a combination therapy involving a VEGF inhibitor (bevacizumab) and an MDM2 inhibitor (Nutlin-3) has been shown to significantly inhibit tumor proliferation and angiogenesis [32]. In summary, MDM2 functions as an oncogene in malignancies and facilitates disease progression and resistance to anticancer treatments through various malignant pathways, as illustrated in Fig. 1.

#### MDM2 provokes immune tolerance in malignancies, resulting in insensitivity to anticancer immunotherapy

MDM2 is widely expressed in various types of malignancies, making it a potential tumor-associated antigen (TAA). Antigen-specific CD8 + autologous T lymphocytes can identify MDM2, and its epitopes can serve as a TAA to activate cytotoxic T lymphocytes (CTLs) that can eliminate tumor cells expressing MDM2 [11, 33]. However, in vivo experimentation has shown that the development of self-tolerance to tumor cells expressing MDM2 can occur among MDM2-specific CTLs [34]. In addition, long-term stimulation by the MDM2 epitope can cause effector CTLs to disappear, which ultimately limits the antigen-specific antitumor response [35]. Furthermore, it was observed that a subset of MDM2-specific T cells managed to evade thymic deletion and remained present in the peripheral T-cell population. Upon exposure to the relevant antigen, these T cells exhibited the ability to initiate cell division but failed to proliferate and expand, leading to a high incidence of apoptosis [36]. Fortunately, it has been established that TCR gene transfer technology can be used to effectively overcome the self-tolerance of autologous T lymphocytes to MDM2, thereby paving the way for a TCR gene transfer-based immunotherapy strategy for treating malignant conditions [34]. Previous research [37] has indicated that high MDM2 expression and low MHC expression are functionally linked to the negative regulation of p53, which plays a role in immune tolerance of cancers [38]. Additionally, the MDM2 inhibitor Nutlin-3 was found to enhance antitumor responses of MDM2-specific T cells by upregulating human leukocyte antigen (HLA) class II expression on tumor cells with the aid of CD4 + T cells [39]. In mice with melanoma, inhibiting MDM2 improved the effectiveness of immune checkpoint inhibitor therapy [40]. Furthermore, it was demonstrated that activating p53 pharmacologically through MDM2 inhibition led to the expression of genes that encode endogenous retroviruses (ERV) [41]. The derepression of ERV activated the ERV-dsRNA-IFN pathway, resulting in the transcription of genes related to antigen processing and presentation, such as β2-microglobulin, HLA-A, HLA-B, and HLA-C [41, 42].

**Fig. 2:**
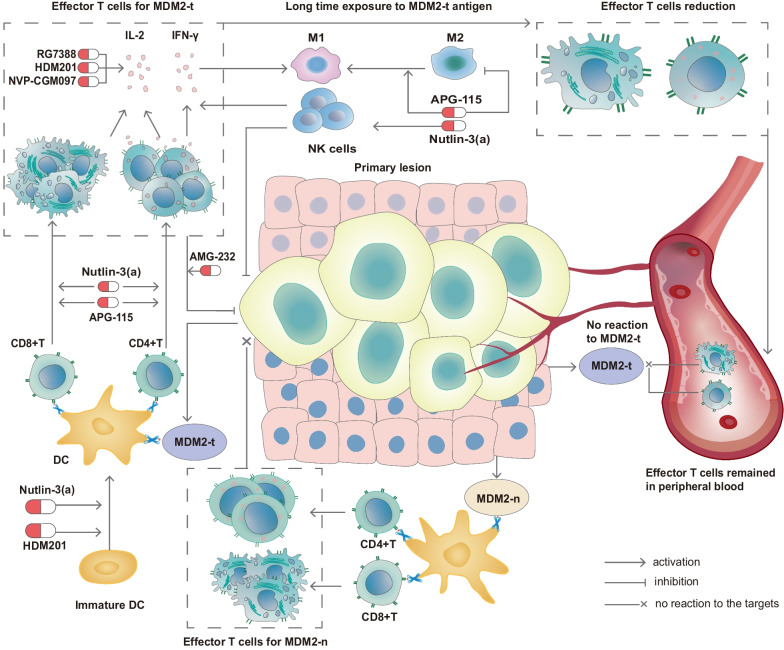
**MDM2 can induce the immune tolerance of malignancies. **Prolonged exposure to tumor MDM2 (referred to as MDM2-t) results in a decrease in the levels of effector T cells that specifically target MDM2-t. Effector T cells targeting MDM2-t, which remain present in peripheral blood, do not exhibit any response to additional MDM2-t restimulation. Furthermore, effector T cells targeting MDM2 that are generated by normal tissue (MDM2-n) do not possess cytotoxic properties toward tumor cells. All of these complex mechanisms contribute to the immune tolerance exhibited toward tumor cells expressing MDM2. The administration of various MDM2 inhibitors can modulate this immune tolerance, which in turn can influence the efficacy of anticancer immunotherapy

Furthermore, the expression of MDM2 is evident in multiple normal tissue types [11], potentially contributing to T lymphocyte central and/or peripheral tolerance, consequently impeding the effective control of tumor growth. Notably, the generation of only low-avidity CTLs targeting a naturally presented human MDM2 peptide epitope that exhibited high affinity toward HLA-A2 molecules was observed. Despite peptide-specific killing, these low-avidity CTLs were insufficient in inducing lysis of human tumor cells that expressed MDM2 [43]. Furthermore, it was revealed that there is a negative association between P53 expression and PD-L1, which serves as a negative regulator of T cell activation. Moreover, the re-establishment of P53 expression through MDM2 inhibition would mitigate the elevated PD-L1 expression in malignant conditions [44]. To provide a comprehensive view of the immune tolerance elicited by MDM2, we created a schematic diagram, illustrated in Fig. 2.

#### MDM2 can modulate the TIME by regulating CD4 + and CD8 + T cells and induce the immune evasion in malignancies

Although CD4 + T cells can recognize the epitope of tumor cell MDM2 and directly eliminate tumor cells [39], immune tolerance still develops. Previous research has shown that MDM2 protein, whether expressed in tumor cells or CD4 + T cells, negatively regulates tumor-infiltrating CD4 + T cells and contributes to immune evasion. The findings indicate that the suppression of MDM2 leads to a notable rise in the serum concentrations of interleukin-2 (IL-2) and interferon-γ (IFN-γ), in addition to an increase in the CD4 + T-cell count. This outcome can be attributed to the upstream OCT3/4, which appears to strengthen the TET1/NRF2/MDM2 pathway, thereby promoting tumorigenesis and immune evasion [45]. In addition, through a P53-dependent mechanism, MDM2 degrades the P53 protein via its E3 ligase function and impairs the P53-enhanced proliferation of CD4 + mature T cells [46]. Additionally, MDM2 has been shown to participate in the negative regulation of CD4 + T-cell activation by inducing the ubiquitination and degradation of the transcription factor NFATc2 [47], which is directly involved in the inhibition of CD4 + T cells in a P53-independent manner. USP15 is classified as a deubiquitinase (DUB) and has been shown to interact with MDM2 directly, stabilizing the MDM2 protein and inhibiting degradation induced by the proteasome system. Subsequently, MDM2 serves as the E3 ligase and facilitates the ubiquitination and accelerated degradation of NFATc2 in naïve CD4 + T cells, ultimately hindering the maturation and activation of CD4 + T cells.

However, the function of MDM2 in CD8 + T cells that have infiltrated malignancies differs considerably from that in CD4 + T cells. Initially, CD8 + T cells were found to exhibit substantially lower MDM2 expression than CD4 + T cells, which was linked to decreased Mdm2 mRNA expression. Correspondingly, the absence of USP15 did not substantially influence NFATc2 activation or cytokine production in naïve CD8 + T cells, which was in stark contrast to the effect observed in naïve CD4 + T cells. This finding accentuates the importance of MDM2 in the inhibition of NFATc2 activation and the induction of cytokine production in T cells [47]. According to a study conducted by other researchers, MDM2 plays a vital role in preserving CD8 + T-cell-driven antitumor immunity by amplifying the STAT5 signaling pathway, which differs substantially from the process of antitumor immunity mediated by CD4 + T cells [48]. The study indicates that degradation of c-Cbl, the E3 ligase responsible for STAT5 ubiquitination and degradation, is facilitated by MDM2, thereby initiating CD8 + T-cell survival and effector function. Nevertheless, this study also highlights the potential of MDM2-targeted therapy in boosting anticancer immunotherapy. The study further revealed that APG115, which inhibits the P53-MDM2 interaction, leads to a dose-dependent upregulation of P53 and MDM2 and promotes T-cell-mediated antitumor immunity while treating cancers. Furthermore, inhibiting MDM2 also directly affected T cells, resulting in increased expression of cytolytic molecules such as perforin and CD107a in CD8 T cells in naïve mice treated with the MDM2 inhibitor compared to mice treated with the vehicle [37]. In mouse tumor models, NVP-CGM097, an MDM2 inhibitor, led to an elevation in the dendritic cell count, the proportion of T cells in tumors and tumor-draining lymph nodes, and the ratio of CD8 + T cells to regulatory T cells in tumors [49]. In the next section, we provide a more in-depth discussion of the critical role of targeted MDM2 therapy in antitumor immunotherapy. Further research is necessary to fully understand the impact of MDM2 on CD8 + T cells.

**Fig. 3:**
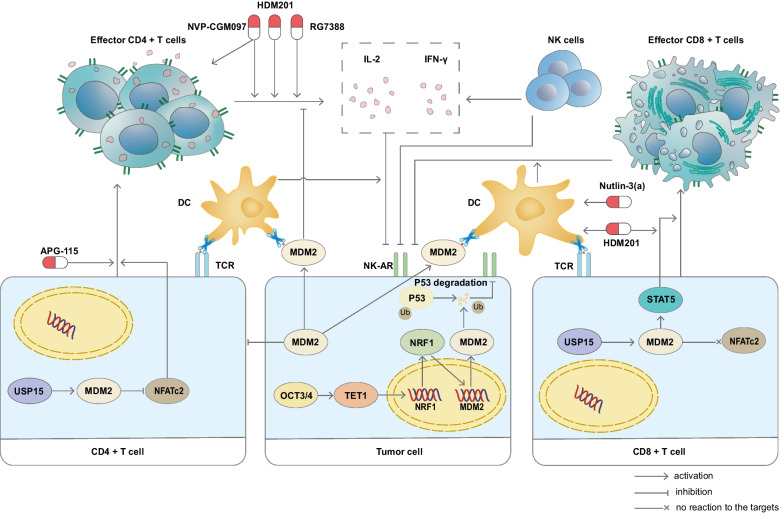
MDM2 can induce the immune evasion of tumor cells. In tumor cells, MDM2 is known to increase the degradation of P53, which consequently results in a decline in the levels of natural killer cell-activating receptors (NK-ARs) and NK-mediated killing. Additionally, MDM2 in these cells can directly impede CD4 + T cells, as well as the production of interleukin-2 (IL-2) and interferon-γ (IFN-γ). Furthermore, MDM2 in CD4 + T cells hinders NFATc2 expression, thereby suppressing the activation of these cells. However, MDM2 in CD8 + T cells can actually stabilize STAT5 and boost their activation. Despite this, APG-115 presents an effective opportunity to upregulate both P53 and MDM2 and thereby enhance the anticancer immunity induced by CD8 + T cells. The figure also demonstrates the effectiveness of MDM2 inhibitors in inhibiting immune evasion induced by MDM2

Based on existing evidence, it is clear that MDM2 plays an important role in the regulation of CD4 + and CD8 + T-cell activation and function, as well as in the modification of the immune checkpoint TIME, as displayed in Fig. 3. Given the crucial role of MDM2 in anticancer immunity, targeted MDM2 therapy presents a promising avenue for salvage treatment in anticancer immunotherapy.

### Targeting MDM2 presents a promising approach to surmounting immunotherapy resistance, thereby offering opportunities for cancer treatment.

Based on a review of published studies [39, 45, 48–53], six types of MDM2 inhibitors have demonstrated a synergistic effect on anticancer immunotherapy in various malignancies. These inhibitors include Nutlin-3(a), NVP-CGM097, HDM201, RG7388, APG-115, and AMG-232. The inhibitors were found to be effective in treating head and neck squamous cell carcinoma (HNSCC), neuroblastoma, bladder cancer, colorectal cancer, breast cancer, and ovarian clear cell carcinoma, as detailed in Table 2. It is important to note that all of the MDM2 inhibitors directly target the interaction of P53 and MDM2. Indisputable preclinical and clinical data demonstrate that targeting MDM2 treatment is a newly discovered approach that can enhance both the effectiveness and safety of cancer therapeutics [54]. Small molecule MDM2 inhibitors effectively sequester region I of the MDM2 protein and thereby prevent its binding to p53 through competitive binding, thus elevating the level of p53 and fostering the activation of the p53 signaling pathway [55, 56]. Consequently, small molecule MDM2 inhibitors can impede the cell cycle, inhibit cell growth, and encourage cell apoptosis [57]. However, there is a need for further research on and discussion of the potential supportive role of MDM2 inhibitors in antitumor immunotherapy. Previous research has suggested that inhibiting MDM2 could lead to the generation of various immune-related cytokines. It has been demonstrated that P53 is essential for maintaining the function of cGAS-STING-TBK1-IRF3 pathway [58], which is responsible for the production of type I interferon [2], and MDM2 inhibition can trigger the production of TNF-α and IFN-γ [37], as well as interleukin-15 (IL-15) production [40]. IL-15 functions as an activator of antitumor CD8 + T cells and natural killer (NK) cells. A recent clinical trial involving patients with metastatic malignancies revealed an increase in the number of NK cells and CD8 + memory T cells following IL-15 treatment [59]. It is worth further discussing whether other mechanisms contribute to the enhancement of immune response induced by MDM2 inhibition.

**Table 2:** Information on MDM2 inhibitors that exhibit synergistic effects on anticancer immunotherapy

Inhibitor	Target	References	Cancer type	Mechanism for the synergistic effect on anticancer immunotherapy
Nutlin-3(a)	MDM2-P53 interaction	[39]	HNSCC	The upregulation of HLA and CD4 + T cells-associated cytotoxicity is facilitated by Nutlin-3
		[50]	NA	Nutlin-3 was found to facilitate the maturation process of DCs and to enhance the proliferation of CD4 + and CD8 + T cells that are stimulated by DCs
		[53]	Neuroblastoma	Nutlin-3a has been observed to increase the expression of NK-ARs, leading to an improvement in the efficacy of NK-mediated tumor cell killing
NVP-CGM097	MDM2-P53 interaction	[44]	Bladder cancer	The administration of NVP-CGM097 has been observed to elevate the serum level of IL-2 and IFN-γ, as well as increase the count of CD4 + T cells
HDM201	MDM2-P53 interaction	[49]	Colorectal cancer	HDM201 has been observed to enhance DCs count and increase the CD8 + T/Treg ratio, as well as elevating IL-2 concentration
RG7388	MDM2-P53 interaction	[49]	Colorectal cancer	RG7388 increases the concentration of IL-2
APG-115	MDM2-P53 interaction	[48]	Colorectal cancer/Breast cancer	The stabilization of T-cell STAT5 and consequent activation of CD8 + T-cell-mediated antitumor immunity is facilitated by APG-115
		[50]	Colorectal cancer	APG-115 suppresses M2 macrophage polarization, increases M1 macrophage polarization, and leads to CD4 + T-cell activation
AMG-232	MDM2-P53 interaction	[52]	Ovarian clear cell carcinoma	AMG-232 sensitizes high MDM2-expressing tumor cells to T-cell-mediated killing

HNSCC, head and neck squamous cell carcinoma; HLA, human leukocyte antigen; IL-2, interleukin-2; IFN-γ, interferon-γ; DCs, dendritic cell; NA, not available; NK-ARs, natural killer cell-activating receptors; Treg, regulatory T cells

Nutlin-3(a) is an extensively researched small molecule inhibitor of MDM2 that has demonstrated antitumor effects by means of direct cytotoxicity and inhibition of proliferation while also modifying the tumor stroma and blood vessels in the tumor microenvironment [60]. Recent studies have uncovered a new role for nutlin-3(a) in regulating the TIME, which in turn governs the response to antitumor immunotherapy. In vitro T cell stimulation co-cultures were employed to demonstrate that nutlin-3, an inhibitor of the MDM2/p53 interaction, enhanced the expression of MHC and costimulatory molecules on dendritic cells. Consequently, this led to an augmentation in the proliferation and activation of the stimulated T cells [51]. Specifically, nutlin-3(a) has been found to enhance HLA expression in tumor cells, resulting in improved recognition and killing of such cells by lymphocytes and inhibition of immune evasion [39]. Moreover, nutlin-3(a) accelerates dendritic cell maturation and subsequently enhances CD4 + and CD8 + T-cellproliferation [45]. Finally, nutlin-3(a) increases the expression of natural killer cell-activating receptors (NK-ARs), which enhances NK-mediated tumor cell killing [53]. Taken together, these findings suggest that nutlin-3(a) represents an effective modulator of the TIME and therefore may increase sensitivity to anticancer immunotherapy. NVP-CGM097 is an MDM2 inhibitor that functions by impeding the MDM2-P53 interaction [61], thereby obstructing tumor cell proliferation. This is achieved through upregulation of the tumor suppressor proteins p53 and p21, with concurrent downregulation of phospho-Rb and E2F1 [62]. Recent studies have revealed that administration of NVP-CGM097 also activates anticancer immunity, leading to an elevation in the serum levels of IL-2 and IFN-γ, in addition to an increase in the CD4 + T-cell count [39]. HDM201, or siremadlin [63], has been shown to have the potential to inhibit tumor proliferation and induce apoptosis by targeting the MDM2-P53 interaction and upregulating P21 expression [64]. Additionally, preclinical tumor models have revealed that combining HDM201 with PD-1/PD-L1 blockade can significantly increase antitumor efficacy by boosting the count of DCs and increasing the CD8 + T/Treg ratio while also elevating the concentration of IL-2 [49]. Similar to the properties of NVP-CGM097 and HDM201, RG7388 is noted for its capacity to increase IL-2 levels, thereby augmenting the immune system’s anticancer responses [49]. The novel MDM2 inhibitor APG-115 is believed to possess a higher affinity for MDM2 than other MDM2-p53 inhibitors, which can result in the inhibition of tumor cell proliferation and an enhancement in radiotherapy sensitivity. By rescuing P53 function and inducing the arrest of cells at the G0/G1 phase, APG-115 is a promising therapeutic agent in the field of oncology [65]. Recent research has revealed that APG-115 also plays an important role in regulating infiltrating immune cells in the TIME. This includes the stabilization of T-cell STAT5 and consequent activation of CD8 + T-cell-mediated antitumor immunity [48]. Additionally, APG-115 suppresses M2 macrophage polarization and promotes M1 macrophage polarization, leading to CD4 + T-cell activation. Combined with data from preclinical tumor models, these findings suggest the potential of APG-115 as a supportive agent for anticancer immunotherapy [50]. Table 2 shows the comprehensive variety of enrolled MDM2 inhibitors, including, AMG-232. Similar to other MDM2 inhibitors, AMG-232 has the capacity to effectively trigger p53 activity, resulting in cell cycle arrest and the inhibition of tumor growth, as demonstrated by a previous study [66]. The impact of AMG-232 on anticancer immunity was further evaluated, and recent research [52] suggests that it enhances the sensitivity of high MDM2-expressing tumor cells to T-cell-mediated killing.

In summary, the utilization of MDM2 inhibitors has clearly demonstrated a considerable impact on the TIME by affecting a variety of mechanisms that target multiple immune cells, such as CD4 + and CD8 + T cells, DCs, NK cells and macrophages. Studies have confirmed the potential therapeutic value of MDM2 inhibitors in conjunction with PD1/PD-L1 blockade for malignancies in preclinical settings. The mechanisms and targets of MDM2 inhibitors in regulating TIME function are further illustrated in Figs. 2 and 3.

MDM2-inhibition has recently been investigated in combination with immunotherapy for various solid tumors. A phase I study is being conducted to test the combination of the MDM2-inhibitor APG-115 and pembrolizumab in patients with metastatic solid tumors (NCT03611868). Additionally, for patients with different cancer types such as colorectal cancer, breast cancer, and renal cell cancer, the anti-PD1 antibody spartalizumab is being tested in combination with siremadlin (NCT04785196). Another phase I/II clinical trial (NCT03787602) for merkel cell carcinoma patients is using KRT-232 (AMG232) with or without anti-PD-1/anti-PD-L1 therapy. These trials demonstrate the significant interest in overcoming immune resistance through MDM2-inhibition.

## Conclusion

MDM2 has been identified as a biomarker of poor prognosis for patients undergoing ICI treatment for malignancies. In particular, patients with MDM2 amplification are at a high risk of developing HPD after anticancer immunotherapy, resulting in a substantial impairment of survival rates. Mechanistically, MDM2 acts as an oncogene in the development of malignancies and can promote tumor cell proliferation and metastasis, directly contributing to disease progression. Furthermore, MDM2 plays a key role in modifying the TIME and influencing immune cells, ultimately mediating immune tolerance and evasion. Fortunately, various MDM2 inhibitors have demonstrated efficacy in alleviating the TIME suppression caused by MDM2. This research indicates promising opportunities for combination therapy with MDM2 inhibitors and anticancer immunotherapy.

## Data Availability

All data and material from this study are available.

## Abbreviations

ICIs: Immune checkpoint inhibitors
anti-PD(L)1: Anti-programmed cell death (ligand) 1
OS: Overall survival
HPD: Hyperprogressive disease
EGFR: Epidermal growth factor receptor
NSCLC: Non-small cell lung cancer
ALK: Anaplastic lymphoma kinase
TIME: Tumor immune microenvironment
LUAD: Lung adenocarcinoma
PFS: Progression-free survival
TTF: Time-to-treatment-failure
ERV: Endogenous retroviruses
VEGF: Vascular endothelial growth factor
PDGF: Platelet-derived endothelial growth factor
TAA: Tumor-associated antigen
CTLs: Cytotoxic T lymphocytes
HLA: Human leukocyte antigen
IL-2: Interleukin 2
IFN-γ: Interferon-γ
DUBs: Deubiquitinases
HNSCC: Head and neck squamous cell carcinoma
NK-ARs: Natural killer cell-activating receptors

## References

[1] Hou H, Sun D, Zhang X (2019). The role of MDM2 amplification and overexpression in therapeutic resistance of malignant tumors. Cancer Cell Int.

[2] Sun D, Qian H, Wang J, Xie T, Teng F, Li J, Xing P (2022). ARID1A deficiency reverses the response to anti-PD(L)1 therapy in EGFR-mutant lung adenocarcinoma by enhancing autophagy-inhibited type I interferon production. Cell Commun Signal.

[3] Leighl NB, Hellmann MD, Hui R, Carcereny E, Felip E, Ahn MJ, Eder JP, Balmanoukian AS, Aggarwal C, Horn L, Patnaik A, Gubens M, Ramalingam SS, Lubiniecki GM, Zhang J, Piperdi B, Garon EB (2019). Pembrolizumab in patients with advanced non-small-cell lung cancer (KEYNOTE-001): 3-year results from an open-label, phase 1 study. Lancet Respir Med.

[4] Reck M, Rodríguez-Abreu D, Robinson AG, Hui R, Csőszi T, Fülöp A, Gottfried M, Peled N, Tafreshi A, Cuffe S, O’Brien M, Rao S, Hotta K, Vandormael K, Riccio A, Yang J, Pietanza MC, Brahmer JR (2019). Updated analysis of KEYNOTE-024: pembrolizumab versus platinum-based chemotherapy for advanced non-small-cell lung cancer with PD-L1 tumor proportion score of 50% or greater. J Clin Oncol.

[5] Mok TSK, Wu YL, Kudaba I, Kowalski DM, Cho BC, Turna HZ, Castro G, Srimuninnimit V, Laktionov KK, Bondarenko I, Kubota K, Lubiniecki GM, Zhang J, Kush D, Lopes G, KEYNOTE-042 Investigators (2019). Pembrolizumab versus chemotherapy for previously untreated, PD-L1-expressing, locally advanced or metastatic non-small-cell lung cancer (KEYNOTE-042): a randomised, open-label, controlled, phase 3 trial. Lancet.

[6] Ready N, Hellmann MD, Awad MM, Otterson GA, Gutierrez M, Gainor JF, Borghaei H, Jolivet J, Horn L, Mates M, Brahmer J, Rabinowitz I, Reddy PS, Chesney J, Orcutt J, Spigel DR, Reck M, O’Byrne KJ, Paz-Ares L, Hu W, Zerba K, Li X, Lestini B, Geese WJ, Szustakowski JD, Green G, Chang H, Ramalingam SS (2019). First-line nivolumab plus ipilimumab in advanced non-small-cell lung cancer (CheckMate 568): outcomes by programmed death ligand 1 and tumor mutational burden as biomarkers. J Clin Oncol.

[7] Horn L, Spigel DR, Vokes EE, Holgado E, Ready N, Steins M, Poddubskaya E, Borghaei H, Felip E, Paz-Ares L, Pluzanski A, Reckamp KL, Burgio MA, Kohlhäeufl M, Waterhouse D, Barlesi F, Antonia S, Arrieta O, Fayette J, Crinò L, Rizvi N, Reck M, Hellmann MD, Geese WJ, Li A, Blackwood-Chirchir A, Healey D, Brahmer J, Eberhardt WEE (2017). Nivolumab versus docetaxel in previously treated patients with advanced non-small-cell lung cancer: two-year outcomes from two randomized, open-label, phase III trials (CheckMate 017 and CheckMate 057). J Clin Oncol.

[8] Shaverdian N, Lisberg AE, Bornazyan K, Veruttipong D, Goldman JW, Formenti SC, Garon EB, Lee P (2017). Previous radiotherapy and the clinical activity and toxicity of pembrolizumab in the treatment of non-small-cell lung cancer: a secondary analysis of the KEYNOTE-001 phase 1 trial. Lancet Oncol.

[9] Zhu AX, Finn RS, Edeline J, Cattan S, Ogasawara S, Palmer D, Verslype C, Zagonel V, Fartoux L, Vogel A (2018). Pembrolizumab in patients with advanced hepatocellular carcinoma previously treated with sorafenib (KEYNOTE-224): a non-randomised, open-label phase 2 trial. Lancet Oncol.

[10] Harrington KJ, Ferris RL, Blumenschein G, Colevas AD, Fayette J, Licitra L, Kasper S, Even C, Vokes EE, Worden F (2017). Nivolumab versus standard, single-agent therapy of investigator’s choice in recurrent or metastatic squamous cell carcinoma of the head and neck (Checkmate 141): health-related quality-of-life results from a randomised, phase 3 trial. Lancet Oncol.

[11] Sun D, Liu D, Liu Q, Hou H (2020). Nivolumab induced hyperprogressive disease in advanced esophageal squamous cell carcinoma. Cancer Biol Ther.

[12] Xiong D, Wang Y, Singavi AK, Mackinnon AC, George B, You M (2018). Immunogenomic landscape contributes to hyperprogressive disease after anti-PD-1 immunotherapy for cancer. iScience..

[13] Gainor JF, Shaw AT, Sequist LV, Fu X, Azzoli CG, Piotrowska Z, Huynh TG, Zhao L, Fulton L, Schultz KR (2016). EGFR mutations and ALK rearrangements are associated with low response rates to PD-1 pathway blockade in non-Small cell lung cancer: a retrospective analysis. Clin Cancer Res.

[14] Haratani K, Hayashi H, Tanaka T, Kaneda H, Togashi Y, Sakai K, Hayashi K, Tomida S, Chiba Y, Yonesaka K (2017). Tumor immune microenvironment and nivolumab efficacy in EGFR mutation-positive non-small-cell lung cancer based on T790M status after disease progression during EGFR-TKI treatment. Ann Oncol.

[15] De Luca A, Carotenuto A, Rachiglio A, Gallo M, Maiello MR, Aldinucci D, Pinto A, Normanno N (2008). The role of the EGFR signaling in tumor microenvironment. J Cell Physiol.

[16] Akbay EA, Koyama S, Carretero J, Altabef A, Tchaicha JH, Christensen CL, Mikse OR, Cherniack AD, Beauchamp EM, Pugh TJ (2013). Activation of the PD-1 pathway contributes to immune escape in EGFR-driven lung tumors. Cancer Discov.

[17] Hong S, Chen N, Fang W, Zhan J, Liu Q, Kang S, He X, Liu L, Zhou T, Huang J (2015). Upregulation of PD-L1 by EML4-ALK fusion protein mediates the immune escape in ALK positive NSCLC: implication for optional anti-PD-1/PD-L1 immune therapy for ALK-TKIs sensitive and resistant NSCLC patients. Oncoimmunology.

[18] Sun D, Zhu Y, Zhu J, Tao J, Wei X, Wo Y, Hou H (2020). Primary resistance to first-generation EGFR-TKIs induced by MDM2 amplification in NSCLC. Mol Med.

[19] Kato S, Ross JS, Gay L, Dayyani F, Roszik J, Subbiah V, Kurzrock R (2018). Analysis of MDM2 amplification: next-generation sequencing of patients with diverse malignancies. JCO Precis Oncol.

[20] Kato S, Goodman A, Walavalkar V, Barkauskas DA, Sharabi A, Kurzrock R (2017). Hyperprogressors after immunotherapy: Analysis of genomic alterations associated with accelerated growth rate. Clin Cancer Res.

[21] Fang W, Zhou H, Shen J, Li J, Zhang Y, Hong S, Zhang L (2020). MDM2/4 amplification predicts poor response to immune checkpoint inhibitors: a pan-cancer analysis. ESMO Open.

[22] Mao S, Zhang J, Guo Y, Zhang Z, Wu Y, Zhang W, Wang L, Geng J, Yan Y, Yao X (2019). Hyperprogression after anti-programmed cell death ligand-1 therapy in a patient with recurrent metastatic urothelial bladder carcinoma following first-line cisplatin-based chemotherapy: a case report. Drug Des Devel Ther.

[23] Bose I, Ghosh B (2007). The p53-MDM2 network: from oscillations to apoptosis. J Biosci.

[24] Gershon TJ, Oren M (1999). Mdm2: the ups and downs. Mol Med.

[25] Wu J, Gou W, Wang Z, Chang H, Li D, Hou W, Liu C (2023). Discovery of the radio-protecting effect of Ecliptae Herba, its constituents and targeting p53-mediated apoptosis in vitro and in vivo. Acta Pharm Sin B.

[26] Feng CJ, Xian QJ, Liu ST (2018). Micro RNA-518 inhibits gastric cancer cell growth by inducing apoptosis via targeting MDM2. Biomed Pharmacother.

[27] Drakos E, Singh RR, Rassidakis GZ, Schlette E, Li J, Claret FX, Ford RJ, Vega F, Medeiros LJ (2011). Activation of the p53 pathway by the MDM2 inhibitor nutlin-3a overcomes BCL2 overexpression in a preclinical model of diffuse large B-cell lymphoma associated with t(14;18)(q32;q21). Leukemia.

[28] Sun D, Feng F, Teng F, Xie T, Wang J, Xing P, Qian H, Li J (2023). Multiomics analysis revealed the mechanisms related to the enhancement of proliferation, metastasis and EGFR-TKI resistance in EGFR-mutant LUAD with ARID1A deficiency. Cell Commun Signal.

[29] Ou M, Xu X, Chen Y, Li L, Zhang L, Liao Y, Sun W, Quach C, Feng J, Tang L (2021). MDM2 induces EMT via the B-Raf signaling pathway through 14-3-3. Oncol Rep.

[30] Inada K, Toi M, Yamamoto Y, Suzuki A, Kurisaki T, Suzuki H, Tominaga T (1996). Immunocytochemical analysis of MDM2 protein expression and its relevance to tumor angiogenesis in primary breast cancer. Oncol Rep.

[31] Venkatesan T, Alaseem A, Chinnaiyan A, Dhandayuthapani S, Kanagasabai T, Alhazzani K, Dondapati P, Alobid S, Natarajan U, Schwartz R, Rathinavelu A (2018). MDM2 overexpression modulates the angiogenesis-related gene expression profile of prostate cancer cells. Cells.

[32] Patterson DM, Gao D, Trahan DN, Johnson BA, Ludwig A, Barbieri E, Chen Z, Diaz-Miron J, Vassilev L, Shohet JM, Kim ES (2011). Effect of MDM2 and vascular endothelial growth factor inhibition on tumor angiogenesis and metastasis in neuroblastoma. Angiogenesis.

[33] Mayr C, Bund D, Schlee M, Bamberger M, Kofler DM, Hallek M, Wendtner CM (2006). MDM2 is recognized as a tumor-associated antigen in chronic lymphocytic leukemia by CD8+ autologous T lymphocytes. Exp Hematol.

[34] Stanislawski T, Voss RH, Lotz C, Sadovnikova E, Willemsen RA, Kuball J, Ruppert T, Bolhuis RL, Melief CJ, Huber C, Stauss HJ, Theobald M (2001). Circumventing tolerance to a human MDM2-derived tumor antigen by TCR gene transfer. Nat Immunol.

[35] Ramírez F, Ghani Y, Stauss H (2004). Incomplete tolerance to the tumor-associated antigen MDM2. Int Immunol.

[36] Bendle GM, Xue SA, Holler A, Stauss HJ (2007). A study of T cell tolerance to the tumor-associated antigen MDM2: cytokines can restore antigen responsiveness, but not high avidity T cell function. PLoS ONE.

[37] Ho JNHG, Schmidt D, Lowinus T, Ryoo J, Dopfer EP, Gonzalo Núñez N, Costa-Pereira S, Toffalori C, Punta M, Fetsch V, Wertheimer T, Rittmann MC, Braun LM, Follo M, Briere C, Vinnakota JM, Langenbach M, Koppers F, Shoumariyeh K, Engel H, Rückert T, Märklin M, Holzmayer S, Illert AL, Magon F, Andrieux G, Duquesne S, Pfeifer D, Staniek J, Rizzi M, Miething C, Köhler N, Duyster J, Menssen HD, Boerries M, Buescher JM, Cabezas-Wallscheid N, Blazar BR, Apostolova P, Vago L, Pearce EL, Becher B, Zeiser R (2022). Targeting MDM2 enhances antileukemia immunity after allogeneic transplantation via MHC-II and TRAIL-R1/2 upregulation. Blood.

[38] Christopher MJ, Petti AA, Rettig MP, Miller CA, Chendamarai E, Duncavage EJ, Klco JM, Helton NM, O’Laughlin M, Fronick CC, Fulton RS, Wilson RK, Wartman LD, Welch JS, Heath SE, Baty JD, Payton JE, Graubert TA, Link DC, Walter MJ, Westervelt P, Ley TJ, DiPersio JF (2018). Immune escape of relapsed AML cells after allogeneic transplantation. N Engl J Med.

[39] Kono M, Kumai T, Hayashi R, Yamaki H, Komatsuda H, Wakisaka R, Nagato T, Ohkuri T, Kosaka A, Ohara K, Kishibe K, Takahara M, Katada A, Hayashi T, Celis E, Kobayashi H, Harabuchi Y (2021). Interruption of MDM2 signaling augments MDM2-targeted T cell-based antitumor immunotherapy through antigen-presenting machinery. Cancer Immunol Immunother.

[40] Langenbach M, Giesler S, Richtsfeld S, Costa-Pereira S, Rindlisbacher L, Wertheimer T, Braun LM, Andrieux G, Duquesne S, Pfeifer D, Woessner NM, Menssen HD, Taromi S, Duyster J, Börries M, Brummer T, Blazar BR, Minguet S, Turko P, Levesque MP, Becher B, Zeiser R (2023). MDM2 inhibition enhances immune checkpoint inhibitor efficacy by increasing IL15 and MHC class II production. Mol Cancer Res.

[41] Zhou X, Singh M, Sanz Santos G, Guerlavais V, Carvajal LA, Aivado M, Zhan Y, Oliveira MMS, Westerberg LS, Annis DA, Johnsen JI, Selivanova G (2021). Pharmacologic activation of p53 triggers viral mimicry response thereby abolishing tumor immune evasion and promoting antitumor immunity. Cancer Discov.

[42] Brummer T, Zeiser R. The role of the MDM2/p53 axis in anti-tumor immune responses. Blood. 2023.

[43] Bendle GM, Holler A, Downs AM, Xue SA, Stauss HJ (2005). Broadly expressed tumour-associated proteins as targets for cytotoxic T lymphocyte-based cancer immunotherapy. Expert Opin Biol Ther.

[44] Cortez MA, Ivan C, Valdecanas D, Wang X, Peltier HJ, Ye Y, Araujo L, Carbone DP, Shilo K, Giri DK, Kelnar K, Martin D, Komaki R, Gomez DR, Krishnan S, Calin GA, Bader AG, Welsh JW (2015). PDL1 regulation by p53 via miR-34. J Natl Cancer Inst.

[45] Mao M, Yang L, Hu J, Liu B, Liu C, Zhang X, Liu Y, Wang P, Li H (2021). OCT3/4 enhances tumor immune response by upregulating the TET1-dependent NRF2/MDM2 axis in bladder cancer. Genomics.

[46] Takemoto S, Trovato R, Cereseto A, Nicot C, Kislyakova T, Casareto L, Waldmann T, Torelli G, Franchini G (2000). p53 stabilization and functional impairment in the absence of genetic mutation or the alteration of the p14(ARF)-MDM2 loop in ex vivo and cultured adult T-cell leukemia/lymphoma cells. Blood.

[47] Zou Q, Jin J, Hu H, Li HS, Romano S, Xiao Y, Nakaya M, Zhou X, Cheng X, Yang P (2014). USP15 stabilizes MDM2 to mediate cancer-cell survival and inhibit antitumor T cell responses. Nat Immunol.

[48] Zhou J, Kryczek I, Li S, Li X, Aguilar A, Wei S, Grove S, Vatan L, Yu J, Yan Y, Liao P, Lin H, Li J, Li G, Du W, Wang W, Lang X, Wang W, Wang S, Zou W (2021). The ubiquitin ligase MDM2 sustains STAT5 stability to control T cell-mediated antitumor immunity. Nat Immunol.

[49] Wang HQ, Mulford IJ, Sharp F, Liang J, Kurtulus S, Trabucco G, Quinn DS, Longmire TA, Patel N, Patil R, Shirley MD, Chen Y, Wang H, Ruddy DA, Fabre C, Williams JA, Hammerman PS, Mataraza J, Platzer B, Halilovic E (2021). Inhibition of MDM2 promotes antitumor responses in p53 wild-type cancer cells through their interaction with the immune and stromal microenvironment. Cancer Res.

[50] Fang DD, Tang Q, Kong Y, Wang Q, Gu J, Fang X, Zou P, Rong T, Wang J, Yang D, Zhai Y (2019). MDM2 inhibitor APG-115 synergizes with PD-1 blockade through enhancing antitumor immunity in the tumor microenvironment. J Immunother Cancer.

[51] Gasparini C, Tommasini A, Zauli G (2012). The MDM2 inhibitor Nutlin-3 modulates dendritic cell-induced T cell proliferation. Hum Immunol.

[52] Sahin I, Zhang S, Navaraj A, Zhou L, Dizon D, Safran H, El-Deiry WS (2020). AMG-232 sensitizes high MDM2-expressing tumor cells to T-cell-mediated killing. Cell Death Discov.

[53] Veneziani I, Infante P, Ferretti E, Melaiu O, Battistelli C, Lucarini V, Compagnone M, Nicoletti C, Castellano A, Petrini S, Ognibene M, Pezzolo A, Di Marcotullio L, Bei R, Moretta L, Pistoia V, Fruci D, Barnaba V, Locatelli F, Cifaldi L (2021). Nutlin-3a enhances natural killer cell-mediated killing of neuroblastoma by restoring p53-dependent expression of ligands for NKG2D and DNAM-1 receptors. Cancer Immunol Res.

[54] Nag S, Zhang X, Srivenugopal KS, Wang MH, Wang W, Zhang R (2014). Targeting MDM2-p53 interaction for cancer therapy: are we there yet?. Curr Med Chem.

[55] Wang S, Zhao Y, Aguilar A, Bernard D, Yang CY (2017). Targeting the MDM2-p53 protein-protein interaction for new cancer therapy: progress and challenges. Cold Spring Harb Perspect Med.

[56] Gupta A, Shah K, Oza MJ, Behl T (2019). Reactivation of p53 gene by MDM2 inhibitors: a novel therapy for cancer treatment. Biomed Pharmacother.

[57] Kojima K, Konopleva M, McQueen T, O’Brien S, Plunkett W, Andreeff M (2006). Mdm2 inhibitor Nutlin-3a induces p53-mediated apoptosis by transcription-dependent and transcription-independent mechanisms and may overcome Atm-mediated resistance to fludarabine in chronic lymphocytic leukemia. Blood.

[58] Ghosh M, Saha S, Bettke J, Nagar R, Parrales A, Iwakuma T, van der Velden AWM, Martinez LA (2021). Mutant p53 suppresses innate immune signaling to promote tumorigenesis. Cancer Cell.

[59] Conlon KC, Lugli E, Welles HC, Rosenberg SA, Fojo AT, Morris JC, Fleisher TA, Dubois SP, Perera LP, Stewart DM, Goldman CK, Bryant BR, Decker JM, Chen J, Worthy TA, Figg WD, Peer CJ, Sneller MC, Lane HC, Yovandich JL, Creekmore SP, Roederer M, Waldmann TA (2015). Redistribution, hyperproliferation, activation of natural killer cells and CD8 T cells, and cytokine production during first-in-human clinical trial of recombinant human interleukin-15 in patients with cancer. J Clin Oncol.

[60] Secchiero P, di Lasio MG, Gonelli A, Zauli G (2008). The MDM2 inhibitor Nutlins as an innovative therapeutic tool for the treatment of haematological malignancies. Curr Pharm Des.

[61] Fang Y, Liao G, Yu B (2020). Small-molecule MDM2/X inhibitors and PROTAC degraders for cancer therapy: advances and perspectives. Acta Pharm Sin B.

[62] Reuther C, Heinzle V, Nölting S, Herterich S, Hahner S, Halilovic E, Jeay S, Wuerthner JU, Aristizabal Prada ET, Spöttl G, Maurer J, Auernhammer CJ (2018). The HDM2 (MDM2) inhibitor NVP-CGM097 inhibits tumor cell proliferation and shows additive effects with 5-Fluorouracil on the p53–p21-Rb-E2F1 cascade in the p53wild type neuroendocrine tumor cell line GOT1. Neuroendocrinology.

[63] Konopleva M, Martinelli G, Daver N, Papayannidis C, Wei A, Higgins B, Ott M, Mascarenhas J, Andreeff M (2020). MDM2 inhibition: an important step forward in cancer therapy. Leukemia.

[64] Jeay S, Ferretti S, Holzer P, Fuchs J, Chapeau EA, Wartmann M, Sterker D, Romanet V, Murakami M, Kerr G, Durand EY, Gaulis S, Cortes-Cros M, Ruetz S, Stachyra TM, Kallen J, Furet P, Würthner J, Guerreiro N, Halilovic E, Jullion A, Kauffmann A, Kuriakose E, Wiesmann M, Jensen MR, Hofmann F, Sellers WR (2018). Dose and schedule determine distinct molecular mechanisms underlying the efficacy of the p53-MDM2 inhibitor HDM201. Cancer Res.

[65] Yi H, Yan X, Luo Q, Yuan L, Li B, Pan W, Zhang L, Chen H, Wang J, Zhang Y, Zhai Y, Qiu MZ, Yang DJ (2018). A novel small molecule inhibitor of MDM2-p53 (APG-115) enhances radiosensitivity of gastric adenocarcinoma. J Exp Clin Cancer Res.

[66] Canon J, Osgood T, Olson SH, Saiki AY, Robertson R, Yu D, Eksterowicz J, Ye Q, Jin L, Chen A, Zhou J, Cordover D, Kaufman S, Kendall R, Oliner JD, Coxon A, Radinsky R (2015). The MDM2 inhibitor AMG 232 demonstrates robust antitumor efficacy and potentiates the activity of p53-inducing cytotoxic agents. Mol Cancer Ther.

